# Corylin alleviated sepsis-associated cardiac dysfunction via attenuating inflammation through downregulation of microRNA-214-5p

**DOI:** 10.1093/toxres/tfae081

**Published:** 2024-06-07

**Authors:** Chunyan Li, Daorong Hou, Yanhong Huang, Yifan Liu, Yong Li, Cheng Wang

**Affiliations:** Department of Noninvasive Electrocardiology, The First Affiliated Hospital of Ningbo University, No. 59 Liuting Street, Haishu District, Ningbo 315000, China; Key Laboratory of Model Animal Research, Animal Core Facility of Nanjing Medical University, Nanjing Medical University, No. 101 Longmian Avenue, Jiangning District, Nanjing 211166, China; Department of Clinical Medicine, The First Clinical Medical College of Nanjing Medical University, No. 101 Longmian Avenue, Jiangning District, Nanjing 211166, Jiangsu, China; Department of Clinical Medicine, The First Clinical Medical College of Nanjing Medical University, No. 101 Longmian Avenue, Jiangning District, Nanjing 211166, Jiangsu, China; Department of Cardiology, The First Affiliated Hospital of Nanjing Medical University, No. 300 Guangzhou Road, Gulou District, Nanjing 210029, China; Department of Cardiology, The First Affiliated Hospital of Nanjing Medical University, No. 300 Guangzhou Road, Gulou District, Nanjing 210029, China

**Keywords:** corylin, sepsis, cardiac dysfunction, inflammation, microRNA-214-5p

## Abstract

**Background:**

Corylin, a natural flavonoid, is isolated from the fruit of *Psoralea corylifolia* L. Nevertheless, the effect of corylin on sepsis-associated cardiac dysfunction is still unclear. The purpose of this study is to determine the role and mechanism of corylin in sepsis related cardiac dysfunction.

**Methods:**

Experiments were carried out on mice with lipopolysaccharide (LPS) or sepsis induced by cecal ligation and puncture (CLP) or myocardial cell sepsis induced by LPS.

**Results:**

Administration of corylin improved cardiac dysfunction induced by LPS or CLP in mice. Corylin inhibited the increases of interleukin-1 (IL)-1β, IL-6 and tumor necrosis factor (TNF)-α in the heart of mice with LPS or CLP. LPS elevated the levels of IL-1β, IL-6 and TNF-α in cardiomyocytes, which were inhibited by corylin treatment. Corylin attenuated the increases of microRNA (miRNA)-214-5p in the heart of mice with LPS, CLP, LPS-treated NRCMs, H9c2 and AC16 cells. Administration of miRNA-214-5p agomiR reversed the improving effects of corylin on the damaged cardiac function and the increases of IL-1β, IL-6 and TNF-α in mice treated with LPS.

**Conclusion:**

These outcomes indicated that corylin improved sepsis-associated cardiac dysfunction by inhibiting inflammation. And corylin inhibited inflammation of sepsis by decreasing miRNA-214-5p. Downregulation of miRNA-214-5p improved sepsis-associated cardiac dysfunction and inhibited inflammatory factors.

## Introduction

Sepsis is a host immune disorder induced by infection which can lead to multiple organ dysfunction syndrome, high incidence rate and mortality rate.^1^^,^^2^ Great progress has been made in the clinical diagnosis and treatment of sepsis, including improvements in pathogen detection technology, development of organ function support and innovation in anti-infection drugs. However, no drug has been approved specifically for sepsis therapy.^3^ No organ is immune to the dysfunction caused by sepsis. Myocardial function is impaired in sepsis according to clinical research^4^ and animal studies.^5^^,^^6^ Inflammatory response enhanced and levels of inflammatory factors increased in sepsis.^7–9^ Coagulation activation initiated by systemic inflammation can cause organ dysfunction, ultimately leading to multiple organ failure and septic death.^10^ Endotoxin, such as lipopolysaccharide (LPS), liberates cytokines from cardiac myocytes.^11^

Traditional Chinese medicine, which has a history of more than 2,000 years, has been widely used in clinical practice,^12^^,^^13^ such as sepsis therapy.^14^*Psoralea corylifolia L.* is the most popular traditional Chinese herb which has been proved to inhibit palmitate-induced neuronal apoptosis,^15^ protect against hepatic disease,^16^^,^^17^ prevent diabetes,^18^ and has anticancer,^19^ antimicrobial^20^ and antioxidant activity.^21^ Corylin is a main flavonoid isolated from *P. corylifolia L.*, and has been proved to reduce the inflammatory reaction of macrophages^22^ and mouse microglia^23^ induced by LPS. Nevertheless, the effect of corylin on sepsis-associated cardiac dysfunction remains unclear.

MicroRNAs (miRNAs) can negatively regulate expression of genes by accelerating mRNA degradation or weakening mRNA translation.^24^ A variety of miRNAs have been identified as potential therapeutic targets or biomarkers for sepsis.^25^^,^^26^ MiRNA-214, acting as tumor suppressor gene and oncogene, regulates differentiation, cell migration, senescence, angiogenesis and virus replication.^27^ MiRNA-214-3p attenuated myocardial dysfunction induced by sepsis in mice,^28^ while miRNA-214-5p aggravated acute kidney damage associated with sepsis in mice.^29^ However, the effect of miRNA-214-5p on sepsis-associated cardiac dysfunction remains unknown.

In summary, the aim of this study was to determine the effects of corylin on cardiac dysfunction and inflammatory response induced by sepsis. We would explore whether corylin improved cardiac inflammation induced by sepsis by regulating miRNA-214-5p, and the effects of miRNA-214-5p on sepsis-associated cardiac dysfunction and inflammatory response.

## Materials and methods

### Animals

Experiments were carried out on 10 week old male C57BL6/J mice (Vital River Biological Co., Ltd, Beijing, China). All procedures were approved by the Experimental Animal Care and Use Committee of Nanjing University, and were complied with the guidelines of the Care and Use of Experimental Animals (NIH publication No. 85-23, 1996). The mice were kept in a temperature controlled room for 12–12 h of light and dark cycle, and were free to standard food and tap water.

### LPS-induced sepsis model and treatment

Septic cardiac dysfunction was induced by intraperitoneal injection of LPS (10 mg/kg, Sigma, MO, USA). Meanwhile, corylin (5, 10, 20 or 40 mg/kg body weight; Sigma) or equivalent volume of vehicle (0.1% DMSO) was intraperitoneally injected into the mice. Echocardiography was performed 12 h after LPS injection and samples were collected.

### CLP-induced sepsis model and treatment

Cecal ligation and puncture (CLP) procedures were performed as previously described. Briefly, prepared skin disinfection and made a median incision in the lower abdomen under 1.5%–2.5% isoflurane anesthesia. Gently removed the cecum from the incision and thread the 4-0 braided silk line through the midpoint between the root of the colon and the end of the cecum. Then, ligated the cecum with a No. 21 needle, removed the needle, squeezed out a small drop of intestinal content, repositioned the cecum and closed the abdomen. The sham-operated (Sham) mice received a similar surgical process, except ligating the cecum with a 21-gauge needle. Meanwhile, corylin (20 mg/kg body weight; Sigma) or equivalent volume of vehicle (0.1% DMSO) was intraperitoneally injected into the mice. Echocardiography was performed 12 h after CLP and samples were collected.

### Echocardiography

Under 1.5%–2.5% isoflurane anesthesia, the left ventricular (LV) function of mice was examined by transthoracic echocardiography (vevo2100, visualsonics, Toronto, Canada) with a 21 MHz probe. Calculated the cardiac ejection fraction (EF) and fractional shortening (FS). All analyzed measurements were the average of three consecutive cardiac cycles.

### Hemodynamic monitoring

The mice were anesthetized with isoflurane (1.5%–2.5%), and a 1.4-F-conductance micromanometer-tip catheter (Millar Instruments, TX, USA) was inserted into the LV chamber throgh the right carotid artery and across the aortic valve. After stabilizing for 20 min, continuously recorded the signals with a conductivity system (Millar Instruments) connected to a Powerlab A/D converter (PL3508, AD Instruments, Australia). The maximum first derivative value of LV pressure (LV + dP/dt_max_), LV volumes in systole (LVVs) and diastole (LVVd) were obtained and analyzed on a PowerLab data collection system (PL3508, AD Instruments).

### Culture and treatment of cardiomyocytes

As mentioned earlier, primary neonatal rat cardiomyocytes (NRCMs) were isolated from neonatal Sprague Dawley (SD) rats aged 1 to 2 days. Excised and digested the hearts in phosphate buffer saline containing pancreatin (Sigma) and collagenase type II (Worthington Biochemical Corp., Lakewood, NJ). Discarded the atria and great vessels, cut the ventricles into small pieces and further digested with collagenase type II and pancreatin. Collected and cultured cells from digestion. Then, 10% fetal bovine serum (GIBCO) was added to the intact Dulbecco modified Eagle medium (GIBCO) for 2–4 h to reduce fibroblasts. And then NRCMs were enriched. Rat cardiomyocyte line H9c2 and human cardiomyocyte line AC16 were obtained from Cell Resource Center of Chinese Academy of Sciences (Shanghai, China). Cultured the cells at 37 °C with 5% CO_2_ and 95% air. LPS (100 ng/mL) was added into the medium to induce sepsis in vitro. Simultaneously, corylin (2.5, 5, 10, or 20 μM) was added into the medium.

### MTT assay for cardiomyocytes viability

Seeded cardiomyocytes at a concentration of 1 × 10^5^ cells/L in a 96-well plate. Treated the cells with various concentrations of corylin (0, 2.5, 5, 10, 20, 40 and 80 μM) for 24 h. The concentrations of corylin were selected based on previous studies.^22^^,^^23^ According to the ability of living cells which utilized thiazole blue and converted it into purple methazan, the viability of cardiomyocytes was measured by the function of living cells which reduced MTT to methazan. Methazan absorbed 570 nm of light and could be measured by microplate reader (VT, BioTek, USA). The results were normalized to the vehicle group.

### Reverse transcription-quantitative PCR (RT-qPCR)

The mice were killed by cervical dislocation with 2.5% isoflurane anesthesia. Shortly after harvest, the heart samples of mice or myocardial cells were frozen with liquid nitrogen and stored at −80 °C until use. Measured the mRNA levels with an RT-qPCR system (Roche Diagnostics). According to the instructions of PrimeScriptTM RT reagent kit, extracted cDNA from RNA through reversing transcription ™RT Master Mix (plateau Biotechnology Co., Ltd, Shanghai, China) with 10 μL random primers. Then measured mRNA levels with Power SYBR Green PCR Master Mix (Thermo Fisher Scientific, Inc.). Amplified all samples in a 384 well plate three times for 40 cycles. Determined the relative gene expression with the 2^−ΔΔ Ct^ method. Primer sequences were shown in Table 1.

**Table 1:** List of utilized primers for qRT-PCR.

Gene	Species	Forward primer	Reverse primer
IL-1β	Mouse	TCACAGCAGCACATCAACAA	TGTCCTCATCCTGGAAGGTC
IL-6	Mouse	GCTACCAAACTGGATATAATCAGGA	CCAGGTAGCTATGGTACTCCAGAA
TNF-α	Mouse	ACGGCATGGATCTCAAAGAC	GTGGGTGAGGAGCACGTAGT
miRNA-214	Mouse	GCAAGGCTATGGCACTTACCTA	CCTGTTGTTACTGGCCCTCA
GAPDH	Mouse	CCTTCCGTGTTCCTACCCC	GCCCAAGATGCCCTTCAGT
U6	Mouse	AAGTATTTC GATTTCTTGGC	AGGTCGGTGTGAACGGATTTG
IL-1β	Rat	ATGGGATAACGAGGCTTATGTG	CAAGGCCACAGGTATTTTGTC
IL-6	Rat	CCTTCCTACCCCAACTTCCA	GAGTTGGATGGTCTTGGTCC
TNF-α	Rat	GCTCCAGAAGTTGCTTGTGC	AACCAGAGGGCTGTTGATGG
miRNA-214	Rat	AGCATAATACAGCAGGCACAGAC	AAAGGTTGTTCTCCACTCTCTCAC
GAPDH	Rat	GGCACAGTCAAGGCTGAGAATG	ATGGTGGTGAAGACGCCAGTA
U6	Rat	GCTTCGGCAGCACATATACTAAAAT	CGCTTCACGAATTTGCGTGTCAT
IL-1β	Human	ATGATGGCTTATTACAGTGGCAA	GTCGGAGATTCGTAGCTGGA
IL-6	Human	ACTCACCTCTTCAGAACGAATTG	CCATCTTTGGAAGGTTCAGGTTG
TNF-α	Human	CGCTCCCAAGAAGACAG	AGAGGCTGAGGAACAAGCAC
miRNA-214	Human	AGCATAATACAGCAGGCACAGAC	AAAGGTTGTTCTCCACTCTCTCAC
GAPDH	Human	AAGGTGAAGGTCGGAGTCAAC	GGGGTCATTGATGGCAACAATA
U6	Human	CAACAGGCTCGTGAAAGACC	GTTCGTCAACCTAGCGCAG

### Elisa

Shortly after collection, samples from heart of mice or cardiomyocytes were frozened in liquid nitrogen and stored at −80 °C until use. Extracted the total protein in the homogenate and measured with a BCA protein assay kit (Beyotime, Shanghai, China). According to the manufacturer’s instructions, determined the levels of interleukin-1 (IL)-1β, IL-6 and tumor necrosis factor (TNF)-α by specific enzyme immunoassay kits (USCN, Wuhan, China).

### Administration of miRNA-214-5p agomiR or antagomiR

To examine the roles of miRNA-214-5p on sepsis, the mice were injected with miRNA-214-5p agomiR or antagomiR (40 mg/kg; RIBOBIO, Guangzhou, China) through tail vein, and were injected with LPS simultaneously. Conducted echocardiography and harvested samples 12 h after LPS injection.

### Hematoxylin-eosin (H&E) staining

The heart was fixed with 4% paraformaldehyde at room temperature for 48 h. Then embedded in paraffin and sliced into 5 μm-thick sections. The sections were examined by H&E staining kit (Service Biological Technology Co., Ltd, Wuhan, China), and were observed under a light microscope (Olympus Corporation, Tokyo, Japan).

### Statistical analyses

The data were expressed as mean ± standard error of mean (SEM). Assessed statistical significance among groups by one-way analysis of variance (ANOVA) through the Bonferroni post-hoc test with GraphPad Prism (Version 7.0; San Diego, CA). Defined statistical significance as *P*-value < 0.05 (two-tail).

## Results

### Corylin alleviated LPS-induced cardiac dysfunction via attenuation of inflammation

**Fig. 1:**
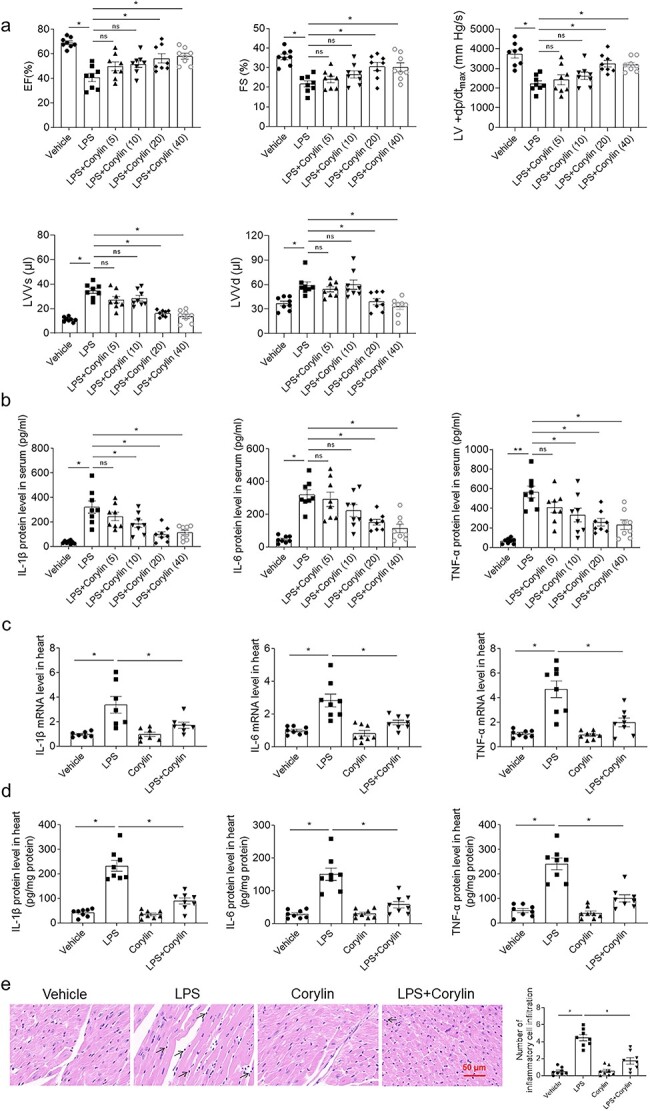
Corylin alleviated cardiac dysfunction induced by LPS through reducing inflammation. a) Corylin could improve the decreases of EF, FS and LV + dp/dt_max_, and inhibited the increases of LVVs and LVVd induced by LPS in mice. b) Corylin inhibited the increases of IL-1β, IL-6 and TNF-α induced by LPS in the serum of mice. c) Corylin inhibited the increases of IL-1β, IL-6 and TNF-α mRNA induced by LPS in the heart of mice. d) Corylin inhibited the enhancements of IL-1β, IL-6 and TNF-α protein induced by LPS in the heart of mice. e) H&E staining of mice heart. The doses of 5 mg/kg, 10 mg/kg, 20 mg/kg and 40 mg/kg were used in a and b. The dose of 20 mg/kg was used in c, d and e. The outcomes were showed as mean ± SEM. N = 8 for each group. ^*^P < 0.05.

Four doses of corylin were selected to determine the effect on sepsis-associated cardiac dysfunction induced by LPS. Decreases of EF and FS in mice were induced by LPS. These decreases were reversed by 20 mg/kg and 40 mg/kg of corylin. 40 mg/kg of corylin didn’t further alleviate the decrease of EF and FS induced by LPS compared with 20 mg/kg of corylin. 5 mg/kg and 10 mg/kg of corylin had no effect on the decreases of EF and FS in mice induced by LPS. The decrease of LV + dp/dt_max_ was improved by corylin (20 mg/kg and 40 mg/kg) treatment. The increases of LVVs and LVVd were inhibited after corylin (20 mg/kg and 40 mg/kg) administration (Fig. 1a). LPS increased the levels of IL-1β, IL-6 and TNF-α in the serum of mice. The increases of IL-1β and TNF-α in the serum of mice induced by LPS were inhibited by administration of 10 mg/kg, 20 mg/kg and 40 mg/kg of corylin. The increases of IL-6 in the serum of mice induced by LPS were suppressed by administration of 20 mg/kg and 40 mg/kg of corylin (Fig. 1b). Selected the dose of 20 mg/kg corylin in the next experiments in vivo. LPS increased the mRNA levels of IL-1β, IL-6 and TNF-α in the heart of mice, which were attenuated by corylin administration (Fig. 1c). LPS increased the protein levels of IL-1β, IL-6 and TNF-α in the heart of mice, which were inhibited by corylin administration (Fig. 1d). The heat sections were performed H&E staining to assess inflammatory cells infiltration. The cardiac slices of sepsis mice showed significant infiltration of inflammatory cells, which could be alleviated by corylin (Fig. 1e).

### Corylin alleviated CLP-induced cardiac dysfunction via attenuation of inflammation

**Fig. 2:**
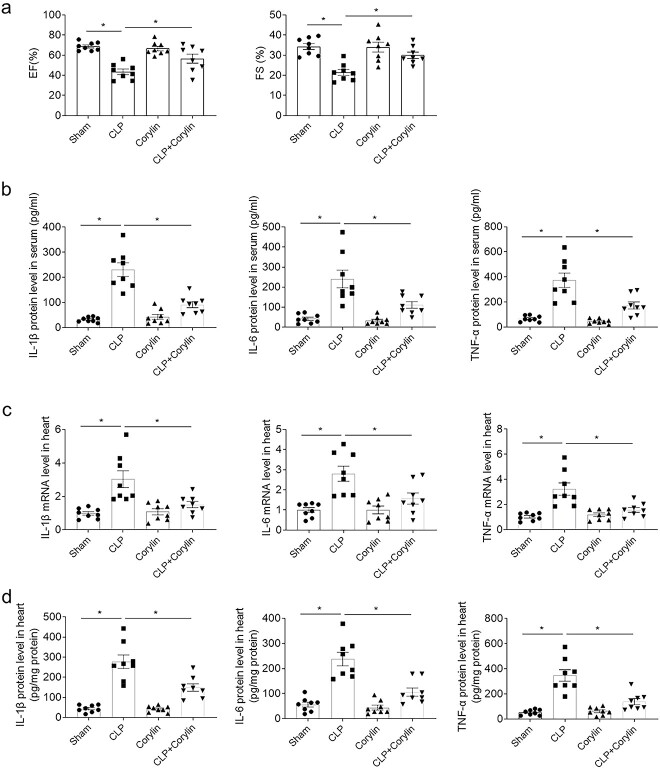
Corylin alleviated cardiac dysfunction induced by CLP through reducing inflammation. a) Corylin improved decreases of EF and FS induced by CLP in mice. b) Corylin inhibited the increases of IL-1β, IL-6 and TNF-α induced by CLP in the serum of mice. c) Corylin inhibited the increases of IL-1β, IL-6 and TNF-α mRNA induced by CLP in the heart of mice. d) Corylin inhibited the increases of IL-1β, IL-6 and TNF-α protein induced by CLP in the heart of mice. The dose of 20 mg/kg was used. The outcomes were showed as mean ± SEM. N = 8 for each group. ^*^P < 0.05.

The decreases of EF and FS of CLP mice were reversed after corylin administration (Fig. 2a). The levels of IL-1β, IL-6 and TNF-α raised in the serum of CLP mice, which were inhibited by corylin (Fig. 2b). The mRNA levels of IL-1β, IL-6 and TNF-α raised in the heat of CLP mice, which were inhibited after administrating of corylin (Fig. 2c). The protein levels of IL-1β, IL-6 and TNF-α raised in the heart of mice induced by CLP, which were attenuated after treatment with corylin (Fig. 2d).

### Corylin alleviated LPS-induced inflammation of cardiomyocytes

**Fig. 3:**
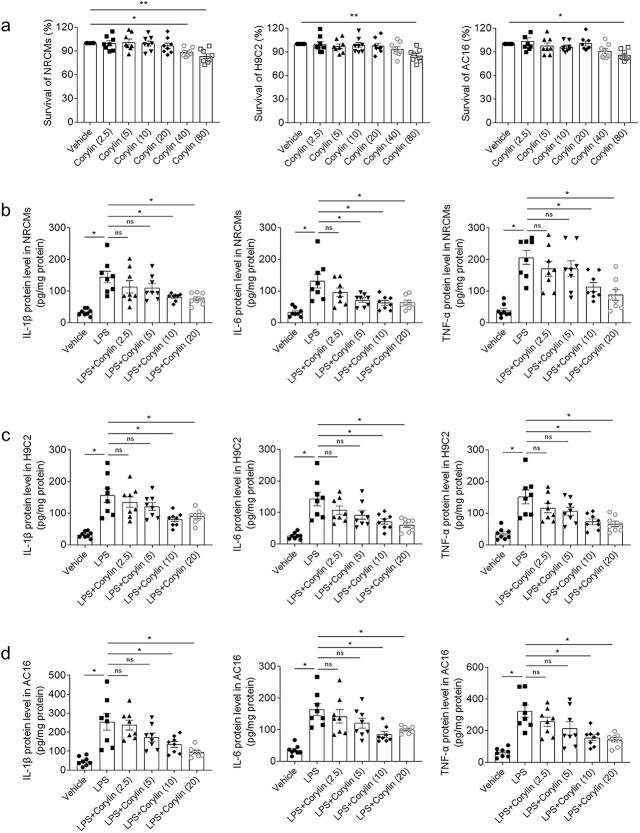
Corylin alleviated inflammation induced by LPS of cardiomyocytes. a) The dose-effects of corylin on cardiomyocyte viability were determined by MTT assay. b) Corylin inhibited the increases of IL-1β, IL-6 and TNF-α protein in NRCMs induced by LPS. c) Corylin inhibited the increases of IL-1β, IL-6 and TNF-α protein in H9c2 cells induced by LPS. d) Corylin inhibited the increases of IL-1β, IL-6 and TNF-α protein in AC16 cells induced by LPS. The doses of 2.5 μM, 5 μM, 10 μM, 20 μM, 40 μM and 80 μM were used in a. The doses of 2.5 μM, 5 μM, 10 μM and 20 μM were used in b, c and d. The outcomes were showed as mean ± SEM. N = 8 for each group. ^*^P < 0.05.

In order to avoid the toxic effect of corylin, we first studied the effect of corylin on cardiomyocyte survival. Analyzed cell viability through the MTT assay. The results showed that there was no significant difference in the NRCMs survival rate treating with corylin 0 ~ 20 μM, H9c2 cells survival rate treating with corylin 0 ~ 40 μM, and AC16 cells survival rate treating with corylin 0 ~ 40 μM (Fig. 3a). In the next studies, 0–20 μM of corylin were selected to determine the attenuating roles of corylin in cardiomyocytes inflammation induced by LPS. LPS increased levels of IL-1β, IL-6 and TNF-α, which were inhibited by 10 μM and 20 μM of corylin in NRCMs (Fig. 3b). The levels of IL-1β, IL-6 and TNF-α elevated in H9c2 cells induced by LPS, which were inhibited by 10 μM and 20 μM of corylin (Fig. 3c). The levels of IL-1β, IL-6 and TNF-α raised in AC16 cells induced by LPS, which were suppressed by 10 μM and 20 μM of corylin (Fig. 3d). 10 μM of corylin was used in the next experiments in vitro*.*

### Corylin inhibited the increases of miRNA-214-5p in the sepsis

**Fig. 4:**
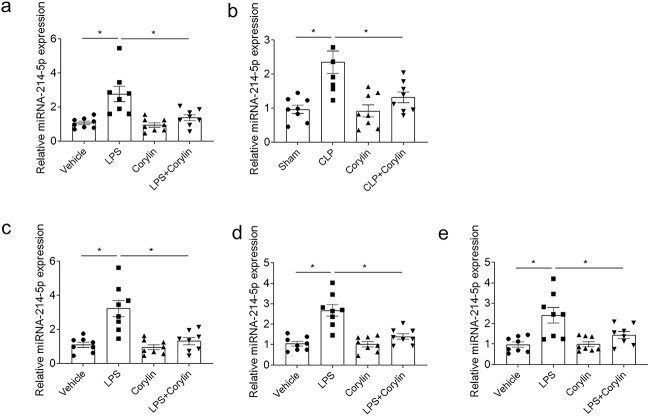
Corylin inhibited the increases of miRNA-214-5p in the sepsis. a) Corylin inhibited the increase of miRNA-214-5p in the heart of mice treated with LPS. b) Corylin inhibited the enhancement of miRNA-214-5p in the heart of CLP mice. c) Corylin inhibited the increase of miRNA-214-5p in the NRCMs induced by LPS. d) Corylin inhibited the increase of miRNA-214-5p in the H9c2 cells induced by LPS. e) Corylin inhibited the increase of miRNA-214-5p in the AC16 cells induced by LPS. The dose of 20 mg/kg was used in a and b. The dose of 10 μM was used in c, d and e. The outcomes were showed as mean ± SEM. N = 8 for each group. ^*^P < 0.05.

The miR-214-5p level raised in the heart of mice treated with LPS, which was inhibited by corylin administration (Fig. 4a). The expression of miR-214-5p increased in the heart of mice with CLP, which was suppressed after treating with corylin (Fig. 4b). LPS increased the level of miRNA-214-5p in NRCMs, which was reversed by corylin (Fig. 4c). LPS increased the level of miRNA-214-5p in H9c2 cells, which was attenuated by corylin (Fig. 4d). The miR-214-5p expression elevated in AC16 cells after LPS stimulation, which was suppressed by corylin treatment (Fig. 4e).

### Upregulation miRNA-214-5p reversed the attenuating effects of corylin on sepsis-associated dysfunction

**Fig. 5:**
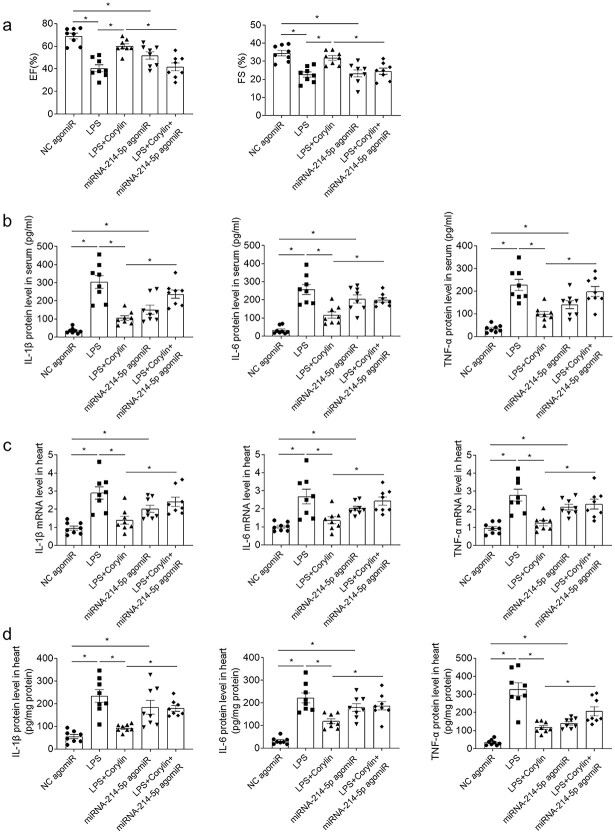
Upregulation of miRNA-214-5p reversed the weakening influences of corylin on sepsis associated with dysfunction. a) Administration of miRNA-214-5p agomiR reversed the improving influences of corylin on the decreases of EF and FS in the mice treated with LPS. b) Administration of miRNA-214-5p agomiR reversed the inhibitory influences of corylin on the enhancements of IL-1β, IL-6 and TNF-α protein levels in the serum of mice treated with LPS. c) Administration of miRNA-214-5p agomiR reversed the inhibitory influences of corylin on the enhancements of IL-1β, IL-6 and TNF-α mRNA levels in the heart of mice treated with LPS. d) Administration of miRNA-214-5p agomiR reversed the inhibitory influences of corylin on the enhancements of IL-1β, IL-6 and TNF-α protein levels in the heart of mice treated with LPS. The dose of 20 mg/kg was used. The outcomes were showed as mean ± SEM. N = 8 for each group. ^*^P < 0.05.

Administration of miRNA-214-5p agomiR reversed the improving influences of corylin on the decreases of EF and FS in mice with sepsis induced by LPS (Fig. 5a). MiRNA-214-5p agomiR administration reversed the attenuating influences of corylin on the enhancements of IL-1β, IL-6 and TNF-α in the serum of mice induced by LPS (Fig. 5b). Treatment with miRNA-214-5p agomiR reversed the inhibiting influences of corylin on the enhancements of IL-1β, IL-6 and TNF-α mNRA in the heart of mice treated with LPS (Fig. 5c). Treatment with miRNA-214-5p agomiR reversed the inhibiting influences of corylin on the enhancements of IL-1β, IL-6 and TNF-α protein in the heart of mice treated with LPS (Fig. 5d).

### Downregulation of miRNA-214-5p alleviated LPS-induced cardiac dysfunction via attenuation of inflammation

**Fig. 6:**
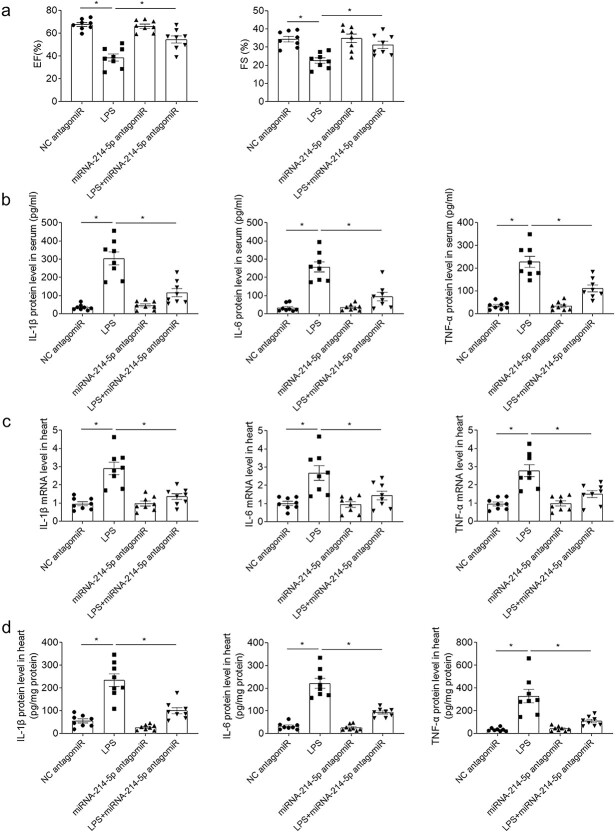
Downregulation of miRNA-214-5p alleviated cardiac dysfunction induced by LPS through reducing inflammation. a) Administration of miRNA-214-5p antagomiR improved the reductions of EF and FS induced by LPS in mice. b) Administration of miRNA-214-5p antagomiR inhibited the increases of IL-1β, IL-6 and TNF-α in the serum of mice induced by LPS. c) Administration of miRNA-214-5p antagomiR inhibited the increases of IL-1β, IL-6 and TNF-α mRNA in the heart of mice induced by LPS. d) Administration of miRNA-214-5p antagomiR inhibited the increases of IL-1β, IL-6 and TNF-α protein in the heart of mice induced by LPS. The dose of 20 mg/kg was used. The outcomes were showed as mean ± SEM. N = 8 for each group. ^*^P < 0.05.

Administration of miRNA-214-5p antagomiR reversed the decreases of EF and FS of mice induced by LPS (Fig. 6a). Treatment with miRNA-214-5p antagomiR inhibited the increases of IL-1β, IL-6 and TNF-α in the serum of mice treated with LPS (Fig. 6b). MiRNA-214-5p administration attenuated the enhancements of IL-1β, IL-6 and TNF-α mRNA levels in the heart of mice induced by LPS (Fig. 6c). MiRNA-214-5p treatment attenuated the enhancements of IL-1β, IL-6 and TNF-α protein levels in the heart of mice with sepsis induced by LPS (Fig. 6d).

### Downregulation of miRNA-214-5p alleviated LPS-induced inflammation of cardiomyocytes

**Fig. 7:**
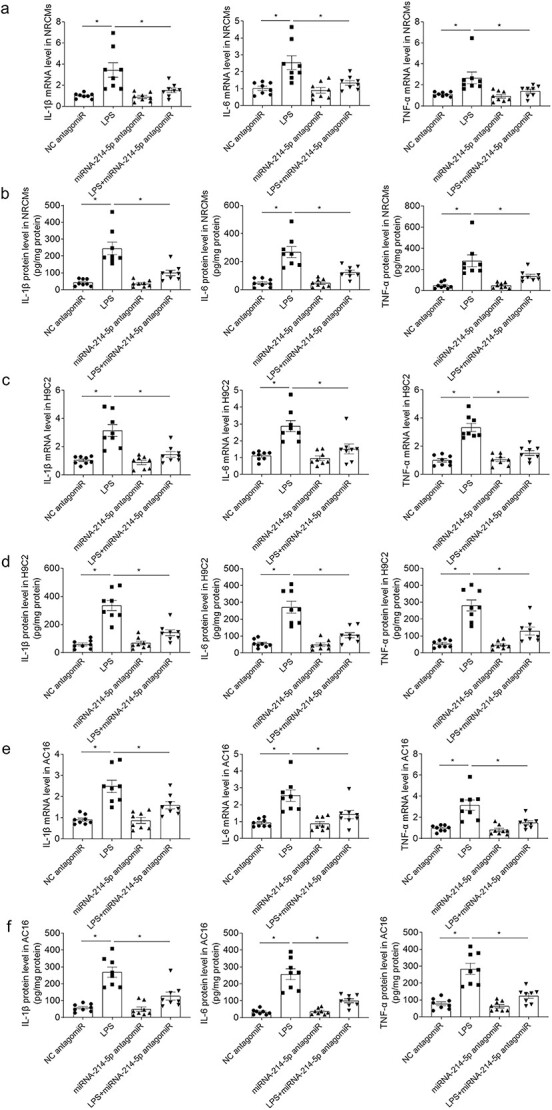
Downregulation of miRNA-214-5p alleviated inflammation induced by LPS of cardiomyocytes. a) Administration of miRNA-214-5p antagomiR inhibited the enhancements of IL-1β, IL-6 and TNF-α mRNA levels in NRCMs induced by LPS. b) Administration of miRNA-214-5p antagomiR inhibited the enhancements of IL-1β, IL-6 and TNF-α protein levels in NRCMs induced by LPS. c) Administration of miRNA-214 antagomiR inhibited the enhancements of IL-1β, IL-6 and TNF-α mRNA levels in H9c2 cells induced by LPS. d) Administration of miRNA-214-5p antagomiR inhibited the enhancements of IL-1β, IL-6 and TNF-α protein levels in H9c2 cells induced by LPS. e) Administration of miRNA-214-5p antagomiR inhibited the enhancements of IL-1β, IL-6 and TNF-α mRNA levels in AC16 cells induced by LPS. f) Administration of miRNA-214-5p antagomiR inhibited the enhancements of IL-1β, IL-6 and TNF-α protein levels in AC16 cells induced by LPS. The dose of 10 μM was used. The outcomes were showed as mean ± SEM. N = 8 for each group. ^*^P < 0.05.

Treatment with miRNA-214-5p antagomiR inhibited the enhancements of IL-1β, IL-6 and TNF-α mRNA levels induced by LPS in NRCMs (Fig. 7a). Treatment with miRNA-214-5p antagomiR suppressed the enhancements of IL-1β, IL-6 and TNF-α protein levels induced by LPS in NRCMs (Fig. 7b). Administration of miRNA-214-5p antagomiR attenuated the enhancements of IL-1β, IL-6 and TNF-α mRNA levels in H9c2 cells induced by LPS (Fig. 7c). Administration of miRNA-214-5p antagomiR suppressed the enhancements of IL-1β, IL-6 and TNF-α protein levels in H9c2 cells induced by LPS (Fig. 7d). Administration of miRNA-214-5p antagomiR attenuated the enhancements of IL-1β, IL-6 and TNF-α mRNA levels in AC16 cells induced by LPS (Fig. 7e). Treatment with miRNA-214-5p antagomiR suppressed the enhancements of IL-1β, IL-6 and TNF-α protein levels in AC16 cells induced by LPS (Fig. 7f).

## Discussion

The novel discoveries of this research were that administration of corylin could improve the cardiac dysfunction of septic mice. The increase of inflammatory response in the heart of mice with sepsis induced by LPS or CLP was attenuated after treating with corylin. The increased level of miRNA-214-5p in septic heart and LPS-treated cardiomyocytes were suppressed by treating with corylin. Downregulation of miRNA-214-5p improved the sepsis-associated cardiac dysfunction and inhibited inflammatory factors.

Sepsis leads to impaired myocardial function and enhanced inflammatory response.^30^^,^^31^ Corylin, a main compound isolated from *P. corylifolia L.*, has been indicated to exhibit various biological properties, such as anti-inflammatory^32^ and anti-tumor effects.^33^ However, the influences of corylin on inflammation and cardiac dysfunction caused by LPS have not been examined. We present found that corylin improved cardiac dysfunction of septic mice. The increases of inflammatory factors in the heart of mice induced by LPS treatment or CLP surgery were suppressed by administration of corylin. These results demonstrate that corylin can improve cardiac dysfunction through attenuating inflammatory response. Corylin may be a drug for heart injury caused by sepsis in the future. In addition, LPS induced the increases of inflammatory factors in cardiomyocytes, which had been supported by a previous study that inflammatory factors could be released from myocytes after LPS stimulation.^7^^,^^34^

Previous studies had found that traditional Chinese medicines alleviated diseases by regulating the expression of miRNAs.^35^^,^^36^ We explored the miR-214-5p levels in the heart of mice with sepsis after treating with corylin. The outcomes indicated that miR-214-5p expressions elevated in the heart of mice with sepsis, which were inhibited by administration of corylin. What’s more, the increases of miR-214-5p in cardiomyocytes induced by LPS were also suppressed by treatment of corylin. These outcomes demonstrate that corylin may improve sepsis by upregulating miR-214-5p expression. However, we still don’t know the mechanism of corylin on downregulation of miR-214-5p expression.

MiRNA-214 could alleviate acute kidney injury in mice with sepsis induced by CLP,^37^ MiRNA-214 expression upregulation could improve myocardial injury induced by sepsis, while miRNA-214 expression downregulation could exacerbate myocardial injury induced by sepsis.^38^ MiRNA-214-3p could alleviate septic myocardial injury in mice with sepsis to attenuate myocardial dysfunction.^28^ On the contrary, miRNA-214-5p aggravated acute kidney injury associated with sepsis in mice.^29^ In the current study, we found that miRNA-214-5p agomiR deteriorated cardiac dysfunction and cardiac inflammation and reversed the alleviatory effects of corylin on the cardiac dysfunction and inflammation in the heart of mice with sepsis. Administration of miRNA-214-5p antagomiR improved cardiac dysfunction associated with sepsis and attenuated heart inflammation caused by sepsis. These results indicate that downregulation of miRNA-214-5p can improve cardiac insufficiency associated with sepsis through decreasing of inflammatory response. Corylin improves heart damage induced by sepsis through downregulating of miR-214-5p expression.

Fibroblast growth factor 9 (FGF9) was proved to be a direct target gene of miR-214, and miR-214 inhibited the tumor-promoting effect of cancer-associated fibroblasts on gastric cancer through targeting FGF9.^39^ Luciferase reporter assay recognized caspase 1 as a target gene of miR-214 in glioma cells.^40^ And miRNA-214-5p could inhibit the proliferation of prostate cancer cells through specifically targeting S0X4.^41^ It is worth to explore the target of miRNA-214-5p on the regulation of LPS-induced cardiac inflammation in the future.

There are many limitations in the present study. Firstly, many factors are involved in sepsis-related cardiac dysfunction except cardiomyocytes damage, including the increase in endothelial reactive oxygen species,^42^ inflammatory changes in vascular endothelium, endocardium leading to microcirculatory changes^43^ and abnormal endothelium-leukocyte interaction for inflammatory cytokines.^44^ In the future, the effects of corylin on endocardium, vascular endothelium and immune cells will be probed. Secondly, corylin does not dissolve in the water. It is unclear whether corylin can be applied by oral.

## Conclusion

Administration of corylin can improve cardiac dysfunction of mice with sepsis through inhibiting inflammatory response. Downregulation of miR-214-5p improves cardiac dysfunction and cardiac inflammation, and increase of miR-214-5p deteriorates cardiac dysfunction and inflammation in the heart of mice with sepsis. Corylin improves the cardiac dysfunction and inflammatory response of mice with sepsis through suppressing the expression of miR-214-5p. Corylin may be a drug for treatment of cardiac dysfunction associated with sepsis in the future.

## Data Availability

All data generated or analyzed during this study are included in this published article.
